# Challenges of transferring models of fish abundance between coral reefs

**DOI:** 10.7717/peerj.4566

**Published:** 2018-04-17

**Authors:** Ana M.M. Sequeira, Camille Mellin, Hector M. Lozano-Montes, Jessica J. Meeuwig, Mathew A. Vanderklift, Michael D.E. Haywood, Russell C. Babcock, M. Julian Caley

**Affiliations:** 1 IOMRC and The UWA Oceans Institute, The University of Western Australia, Crawley, Western Australia, Australia; 2 Australian Institute of Marine Science, Townsville, Queensland, Australia; 3 The Environment Institute and School of Biological Sciences, The University of Adelaide, Adelaide, South Australia, Australia; 4 Indian Ocean Marine Research Centre, CSIRO Oceans and Atmosphere, Crawley, Western Australia, Australia; 5 Centre for Marine Futures and School of Biological Sciences, The University of Western Australia, Crawley, Western Australia, Australia; 6 Dutton Park, CSIRO Oceans and Atmosphere, Brisbane, Queensland, Australia; 7 School of Mathematical Sciences, Queensland University of Technology, Brisbane, Queensland, Australia; 8 Australian Research Council Centre of Excellence for Mathematical and Statistical Frontiers, Brisbane, Queensland, Australia

**Keywords:** Great Barrier Reef, Generalized linear mixed-effects modelling, Ningaloo Reef, Species distribution models, Underwater visual counts

## Abstract

Reliable abundance estimates for species are fundamental in ecology, fisheries, and conservation. Consequently, predictive models able to provide reliable estimates for un- or poorly-surveyed locations would prove a valuable tool for management. Based on commonly used environmental and physical predictors, we developed predictive models of total fish abundance and of abundance by fish family for ten representative taxonomic families for the Great Barrier Reef (GBR) using multiple temporal scenarios. We then tested if models developed for the GBR (reference system) could predict fish abundances at Ningaloo Reef (NR; target system), i.e., if these GBR models could be successfully transferred to NR. Models of abundance by fish family resulted in improved performance (e.g., 44.1% <*R*^2^ < 50.6% for Acanthuridae) compared to total fish abundance (9% <*R*^2^ < 18.6%). However, in contrast with previous transferability obtained for similar models for fish species richness from the GBR to NR, transferability for these fish abundance models was poor. When compared with observations of fish abundance collected in NR, our transferability results had low validation scores (*R*^2^ < 6%, *p* > 0.05). High spatio-temporal variability of patterns in fish abundance at the family and population levels in both reef systems likely affected the transferability of these models. Inclusion of additional predictors with potential direct effects on abundance, such as local fishing effort or topographic complexity, may improve transferability of fish abundance models. However, observations of these local-scale predictors are often not available, and might thereby hinder studies on model transferability and its usefulness for conservation planning and management.

## Introduction

Understanding patterns of species’ abundance and their causes remains a central challenge to assist ecological management (Stuart-Smith et al., 2013; Atwood et al., 2015), particularly in marine ecosystems (e.g., Worm et al., 2006). Obtaining reliable abundance estimates for marine fishes can be challenging (Pauly, Hilborn & Branch, 2013) as abundances can vary rapidly and substantially through time and space (Caley et al., 1996; Rana et al., 2014). Marine surveys can also be prohibitively expensive rendering many marine systems severely under-surveyed, or when they are possible, estimates are often constrained by the efficiency of surveys (Reynolds, Thompson & Russell, 2011). This situation is exemplified on coral reefs where underwater visual censuses (UVCs) are commonly used to estimate fish abundances. The application of UVCs is constrained in space and time by the cost of field expeditions and by bottom time and depth limits for divers. These censuses do, however, have the advantage of estimating abundances directly (Halford & Thompson, 1996), including exploited (e.g., lutjanids and lethrinids) and non-exploited species (e.g., pomacentrids). They have been successfully used for estimating fish distributions and population trends in coastal ecosystems (Willis, Millar & Babcock, 2003; Stuart-Smith et al., 2013; among many others), and UVC-estimated fish abundances have served as input for predictive models aiming to estimate abundances at the scale of entire reef ecosystems using broad-scale predictors (e.g., Mellin et al., 2010).

Predictive models can assist identifying affinities of species or individuals for particular environmental conditions and thereby predict their patterns of spatial distribution. By being able to provide such information, predictive models can also help fill knowledge gaps essential for coral reef management (Fisher et al., 2011b). However, the robustness (degree of model fit) of predictive models of fish abundances can vary substantially. While relatively robust predictions of abundance have been obtained for single species (up to ∼70% deviance explained) (e.g., Young & Carr, 2015), considerably less variation has been explained for total fish abundances (∼35%) (e.g., Mellin et al., 2010) or even less for some functional groups (e.g., 16% for piscivores; Pittman, Costa & Battista, 2009).

Because abundance is an important and commonly used ecological metric, having readily available spatial predictions of fish abundances in situations where direct estimates are currently unavailable could be highly beneficial. For example, such information could be used to support stock assessments or guide the establishment of management strategies (Bejarano, Mumby & Sotheran, 2011). In the absence of predictive models, or data with which to build them for a particular location, successful transfer of a pre-existing model built for a data-rich location could be of considerable utility. This procedure of using an existing model to derive predictions for a new location is defined as model transfer*,* with model transferability being the model’s ability to transfer to new locations (Sequeira et al., 2018). Indeed, a recent study (Sequeira et al., 2016) demonstrated the transferability of predictive models of fish species richness between distant coral reefs. However, species richness and fish abundance are two distinct ecological metrics, with inherent differences in spatio-temporal variability caused by different processes. While species richness can be informative for conservation management in the design of marine protected areas, abundance models can provide relevant information for the management of fisheries in stock assessments and setting spatially explicit catch thresholds (Bejarano, Mumby & Sotheran, 2011). Here, we explore the transferability of models for the more variable metric fish abundance considering the same two widely separated reefs recently investigated by Sequeira et al. (2016): the Great Barrier Reef (GBR), a relatively well-studied reef system located off the northeast coast of Australia, and Ningaloo Reef (NR), a fundamentally different and less-studied reef, located off the northwest coast of Australia. In this study, we examine the effects of annual variability in fish counts by accounting for the number of years when fish were surveyed. We investigate a number of scenarios with subsets of data collected in different years and also covering a range of 1 or 4 years at a time. The latter multi-year scenarios allow averaging of inherently variable fish abundance estimates. We also investigate potential effects associated with fishing effort by considering ten representative fish families separately including the exploited fish families Lethrinidae and Lutjanidae. The number of species within each fish family also varies (e.g., Pomacentridae comprises several hundred species, while Zanclidae only one species). Therefore, examining these ten fish families separately allows testing for effects associated with different degrees of abundance variability within and among fish families. We then provide an overview of the difficulties of modelling fish abundances and highlight some of the issues that will need to be overcome before such models can be successfully transferred.

## Materials & Methods

### Data collection and pre-processing

#### Fish counts

We used reef fish UVC data collected from six different sectors of the GBR: Cooktown/Lizard Island, Cairns, Townsville, Whitsunday, Swains, Capricorn Bunkers (Fig. S1). The same 133 sites were surveyed regularly over a decade (2003–2013, but only during odd-numbered years after 2005) by the Long-Term Monitoring Program of the Australian Institute of Marine Science (AIMS; Sweatman et al., 2008). Fish were counted along five, 50 × 5 m (50 × 1 m for small, sedentary species) transects per site, three sites per reef (including reef slope and reef flat) on 46 reefs distributed across three shelf positions (inner, mid and outer) (Halford & Thompson, 1996). To ensure no systematic biases in the fish counts by different observers, calibration exercises were completed annually by all divers (Halford & Thompson, 1996). We pooled fish abundances per unit area (i.e., densities per 250 m^2^) across transects within sites to estimate total fish abundances (*N*_total_) and also by fish family (*N*_fam_) for: Acanthuridae, Chaetodontidae, Labridae, Lethrinidae, Lutjanidae, Pomacentridae, Scaridae, Serranidae, Siganidae, and Zanclidae (for a comprehensive list of surveyed species refer to Sequeira et al., 2016).

We then partitioned this dataset of fish abundance estimates collected on the GBR into a series of scenarios to explore the influence of different input datasets on the transferability of GBR models of fish abundance to NR. One of our objectives was to test if better transferability could be obtained by using data collected in the same year at both locations, in any single year, or in an averaged set of years when data from only a single year are available for the location to where the model is being transferred. In our scenarios, we varied (i) the length of the subset of data used in each scenario (i.e., including only 1 or 4 years of data) to account for annual variability, which is averaged-out in multi-year scenarios allowing to test if such variability compromises prediction and transferability and whether longer time series may provide better results; (ii) survey years (2003–2007, 2007–2013, 2007, and 2013) to account for effects of temporal variability (i.e., old versus more recent) and to test for any potential effects associated with differences in survey times between the reference and the target system; and (iii) transect size by downscaling the GBR data from 50 m to 25 m (resolution of dataset available at NR) to assess how a mismatch in data resolution between the reference and target systems might also affect model transferability. In all, we tested seven different scenarios.

Our downscaling procedure consisted of two steps. First, we used all transects within the same site to calculate the expected average increase in the number of individuals (N) with the addition of the area surveyed in each added transect (T) for each site (S) (i.e., to estimate N at S based on the areas sampled: T1, T1 + T2, T1 + T2+ T3, T1 + T2+ T3 + T4, and T1 + T2+ T3 + T4 + T5). Doing this procedure per site allowed us to account for the inherent spatial differences observed across sites within reefs. To quantify the increase in abundances with area sampled, we repeated this calculation multiple times by randomising the sequence at which the data from each transect was added. This random procedure allowed the calculation of an average increase in N for each transect added within the same site. Second, we subtracted from each transect, the ratio of the calculated average increase in N with the difference in transect length between each reef system (50/25 *m* = 2), i.e., Transect_*N*_–(estimated average increase/2). This downscaling procedure was only applied to the GBR data and assumed that the increase in the number of individuals per transect sampled would only be similar among all transects within the same site (i.e., with no assumption made beyond the reef scale). The final set of seven scenarios examined here included data collected in 2003–2007 (scenario A), in 2007–2013 (B and C), in 2007 (D and E), and in 2013 (F and G) with scenarios C, E and F including downscaled data, as described above. All models were tested for each of these scenarios.

For NR, we used reef fish UVC data from 81 sites on NR (Fig. S1) collected by the Commonwealth Scientific and Industrial Research Organisation (CSIRO). These data were collected in 2013 using a similar procedure to that used in the GBR, but with shorter sets of three transects of 25 × 5 m (25 × 1 m for small, sedentary species) per site.

#### Environmental predictors

Large scale predictors including nutrient inputs (such as NO_3_, which influences fish abundance through increasing local primary productivity), and sedimentation (which is relevant for some species feeding behaviour), have previously been suggested as important predictors of reef fish communities (Pinca et al., 2012). Similarly, water temperature affects the aerobic performance of reef fishes with effects being trait- and species dependent (Mellin et al., 2016), and sea surface temperature has been shown to contribute to the best performing models for predicting fish abundance (Mellin et al., 2010 but see Barneche et al., 2016). Consequently, we used large-scale environmental predictors available at a national scale in Australia (0.01° resolution) (http://www.marinehub.org), including sea surface temperature (*SST*), salinity, nutrients, light (*K490*_*av*_), depth (as proxy for habitat), oxygen, and sediment characteristics including percentages of carbonates, gravel, sand and mud (Table 1). To account for geographical effects in the distributional patterns of reef fish, we also included two spatial predictors: the shortest distances to coast (*coast*) and to the outer limit of the reefs (*barrier*), which have been used to successfully predict their species richness and abundances (Mellin et al., 2010; Sequeira et al., 2016). We calculated these distances for each sampled site and node on the 0.01° national grid using the *Near* tool in ArcGIS10.1 (ESRI, Redlands, CA, USA) and an equidistant cylindrical coordinate system. For NR, we also used hyperspectral bathymetric data (Heyward, 2006). We then assigned each site to the closest node on the 0.01° national grid and used the environmental predictors corresponding to these locations.

**Table 1:** Models relating coral reef fish abundance (N) to spatial and environmental properties in the Great Barrier Reef (GBR) and Ningaloo Reef (NR). *Coast*: distance to coast; *barrier*: distance to the outer limit of the reef; *crbnt*: percentage of carbonates; *gravel*, *sand*, and *mud* also represented as percentage and derived from the Marine Sediment Database (MARS; available at npm.mars.search) (Passlow et al., 2005; Mathews, Heap & Woods, 2007); *NO_3_*: nitrate, *PO_4_*: phosphate, *SI*: silicate, *O_2_*: dissolved oxygen, *Sal*: salinity, all represented as mean concentrations and derived from the CSIRO Atlas of Regional Seas (CARS; available at: http://www.marine.csiro.au) (Dunn & Ridgway, 2002; Ridgway, Dunn & Wilkin, 2002) *SST*: annual sea surface temperature derived from the NASA standard monthly data products from the Advanced Very High Resolution Radiometer (AVHRR) Pathfinder V5; *Chla*: chlorophyll *a*, and *K490*: coefficient of light attenuation at 490 nm derived from the ocean colour standard monthly data products from the Sea-viewing Wide Field-of-view Sensor (SeaWiFS) and Moderate Resolution Imaging Spectroradiometer satellite (MODIS) from the National Aeronautics and Space Administration (NASA); subscript *av*: average. All predictors were mean centred, and *coast*, *barrier*, *depth*, and *SST*_*av*_ were included as quadratic terms in a second-order polynomial function. Sediment variables were mostly collinear and were therefore included in separate models (4 –6). Bold face indicates predictors not included in both systems (due to collinearity between variables observed in NR).

Model	Predictor category	GBR model	NR model
1	Full model	*coast + barrier + slope****+ NO_3_***_**av**_	*coast + barrier + slope*
2	Distance to domain boundaries	*coast + barrier*	
3	Physical predictors including range of depths	*depth + slope*	
4	Sediment characteristics	***crbnt****+ gravel*	*gravel*
5	Sediment characteristics	*sand*	
6	Sediment characteristics	*mud*	
7	Nutrients	*NO_3_*_*av*_*+ PO_4_*_*av*_*+ SI*_*av*_	
8	Oxygen and salinity	*O_2_*_*av*_*+ Sal*_*av*_	
9	Productivity	*Chla*_*av*_	
10	Temperature	*SST*_*av*_	
11	Light availability	*K490*_*av*_	
12	Null model	*1*	

Our model set included different combinations of these environmental predictors, avoiding inclusion of correlated predictors in the same model (Table 1), and accounted for second order polynomials for bathymetric features (*depth*), distances to domain boundaries (*coast* and *barrier*) and sea surface temperature (*SST*). To the extent permitted by the computational demands of the modelling procedures used here (i.e., negative binomial with mixed effects, as described below), we kept the model set as similar as possible to that used in the previously published study of the transferability of species richness models (Sequeira et al., 2016). We did this to facilitate better comparisons of the overall results obtained here with those obtained for species richness. We considered the same set of models for all scenarios for the GBR and also for NR (Fig. S2). All predictors were centred prior to modelling to ease interpretation of resulting coefficient estimates. Estimates of *rugosity,* the variation in the height of the substrate quantified along each transect by the absolute horizontal distance covered by 10 m of light chain moulded to the contours of the substratum, were also available at NR for each sampled transect, but not for the GBR. These rugosity values at NR were included in an additional model with a quadratic term for this predictor to account for a potential best range of rugosity values for higher fish abundance. This additional model was used to get an indication of how much more the NR models could be improved by including local topographical fine-scale predictors that can directly influence fish abundance (e.g., Caley & StJohn, 1996; Wilson, Graham & Polunin, 2007).

### Modelling approach

#### Reference models for GBR and NR

Using the resulting counts for fish abundances (*N*_total_ and *N*_fam_), we developed generalised linear models (GLM) for each ecosystem (GBR and NR), using a negative binomial distribution with a log-link function with the glm.nb() function from the package MASS in R (R Core Team, 2016). Due to spatial autocorrelation of the residuals from the GBR model (Moran’s I > 0.5 at first lag), we developed a generalised linear mixed-effects model (GLMM using the glmer.nb() function from the R package lme4) for the GBR by including *reef* as a random effect. Inclusion of this random effect was also necessary because several sites fell within the 0.01° resolution that characterizes the environmental layers. Thus sites can be considered pseudo replicates at this resolution, which can be addressed with the use of a ‘reef’ random effect (Mellin et al., 2010; Mellin et al., 2012). We used a negative binomial distribution to account for the large dispersion observed in fish abundances (9.2 <dispersion statistic <23.1 calculated after a test model run using a GLMM with a Poisson distribution). We used the weight of the Akaike’s information criteria corrected for small sample sizes (*wAICc*) (Burnham & Anderson, 2004) to rank models, and the marginal ${R}_{m}^{2}$ and conditional ${R}_{c}^{2}$ (Nakagawa & Schielzeth, 2013) to assess their performance. We also calculated the mean effect size for each predictor following the method described in Sequeira et al. (2014). We assessed the correlation between observed and predicted values on sampled sites using the *R*^2^ and *p*-values from a linear regression between the two sets of values to allow direct comparison between all modelling results (reference and transferred models). We then used the models to predict *N*_total_ and *N*_fam_ at the scale of the entire GBR and NR separately. We used a 10-fold cross-validation (Davison & Hinkley, 1997) for estimating prediction errors for each model, as well as the model-averaged prediction error (i.e., across the entire model set) (Table 1). We checked for spatial autocorrelation in model residuals using the *sp.correlogram* function from the R package *spdep* (Bivand, 2013) to calculate Moran’s *I* (Diggle & Ribeiro, 2007). We repeated this procedure for all seven scenarios in the GBR and for NR, separately. We then tested the transferability of the GBR model to NR by transferring the *N*_total_ and *N*_fam_ models corresponding to each GBR scenario to NR.

#### Transferred models from the GBR to NR

Assuming stationarity in species abundance distribution across both ecosystems, we assessed the transferability of the GBR models to NR using *R*^2^ and *p*-values of a linear regression between the observed values for NR and the resulting predictions from the transferred model. We then compared the predictions from the transferred models with those obtained from the model developed for NR. We made the latter comparison following Sequeira et al. (2016), and using (i) the mean direct validation errors of each transferred GBR scenario against observations for NR, (ii) the mean absolute difference between values obtained by the transferred and reference models on a grid-cell basis, (iii) the percentage of grid-cells where the predictions from the transferred models differed by a small amount (≤15%) from the predictions from the reference NR model, and (iv) the *N* patterns predicted for NR by both the reference and transferred models after rescaling the predictions from the transferred models to the same maximum and minimum as the reference predictions.

## Results

### Models for GBR (*N*_total_ and *N*_fam_)

**Figure 1:**
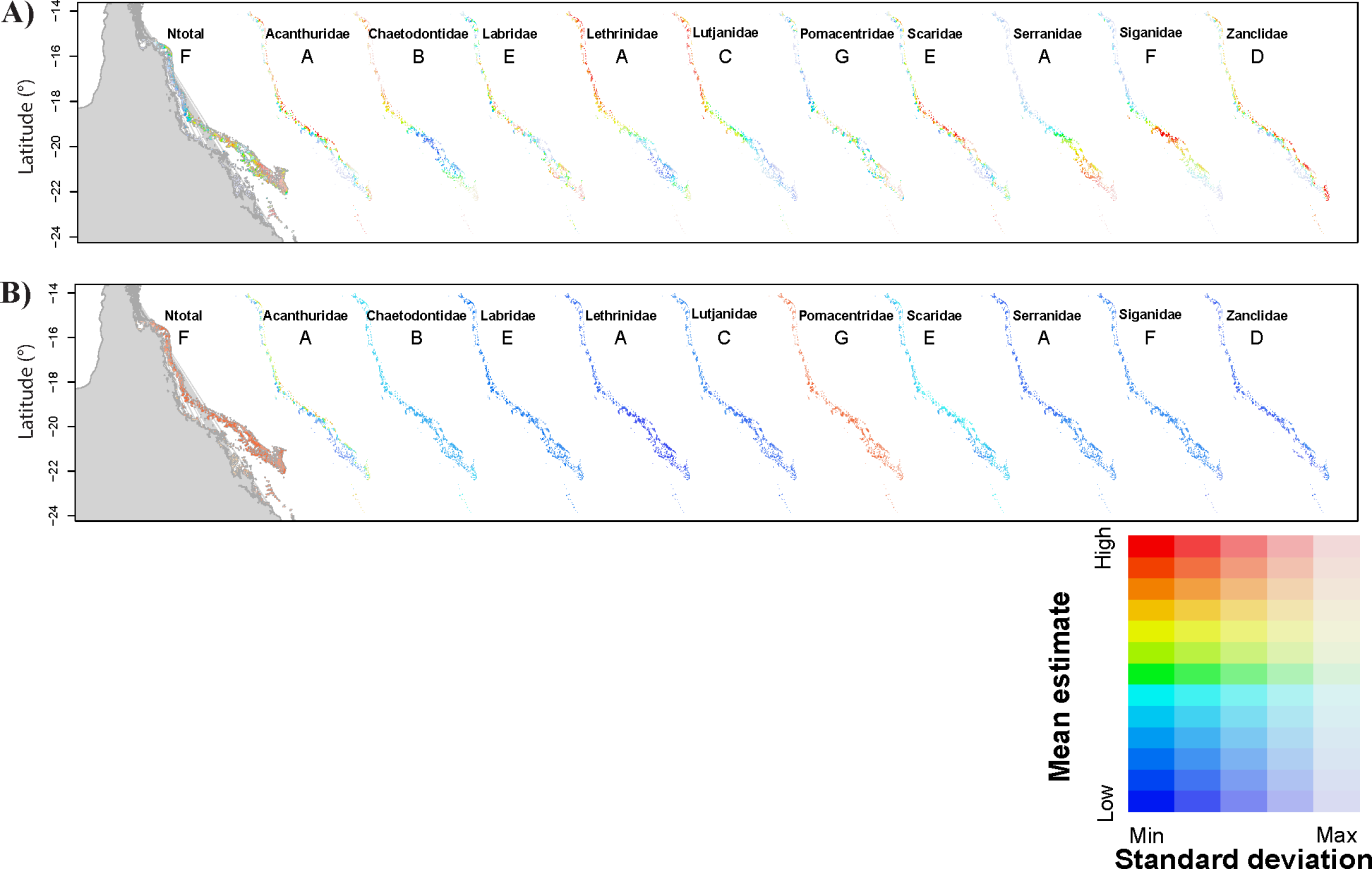
Predictions of total fish abundance (*N*_total_) and fish abundance by fish family to the Great Barrier Reef by the GBR models. Results for each family shown for a representative scenario only (indicated by letter from A to G). Refer to Fig. S1, for predictions from all GBR scenarios. (A) showing maximum and minimum values for each map independently, and (B) reflecting ranges of values across all maps highlighting Pomacentridae as the most abundant fish family, but also with large prediction error (refer to Tables 2 and 3). Predicted minimum and maximum abundance values for each scenario shown here were: 173–958 (*N*_total_), 3–99 (Acanthuridae), 25–51 (Chaetodontidae), 4 –13 (Labridae), 2–6 (Lethrinidae), 2–11 (Lutjanidae), 273–725 (Pomacentridae), 10–50 (Scaridae), 2–8 (Serranidae), 1–10 (Siganidae), and 1–8 (Zanclidae). Grey area represents Queensland in the Northeast of Australia, longitude: 141° to 153°E.

The *N*_total_ models for the GBR resulted in *R*^2^ ranging from 9.0–18.6% across all scenarios, with significant correlations between predictions and observations (*p* ≤ 0.002) (Table 2). Model rankings were similar across all scenarios (Table 2, A–G) with the highest *wAICc* obtained for model 7 (nutrients) but sometimes shared with models 3 (depth), 4 (sand) or 5 (gravel). Most important predictors commonly included *NO*_3_. ${R}_{\mathrm{m}}^{2}$ was ∼20% for the best model in all scenarios while ${R}_{c}^{2}$ was close to 100% (>98.5% for all *N*_total_ models in all scenarios). Cross-validation errors varied between 12.8–22.5% with predicted ranges generally overestimating minimum observed fish abundances in the GBR but maximum values close to the observed for most scenarios (Table 2). The predicted patterns of *N*_total_ for the GBR were also similar for all scenarios with generally greater abundances predicted in two main areas of the southern sections of the GBR (Fig. 1— *N*_total_ and Fig. S3).

**Table 2:** Modelling results for the Great Barrier Reef (GBR) scenarios predicting total fish abundance (*N*_total_) to the GBR and to Ningaloo Reef (NR). Observed *N*: observed fish abundance; Top model: the best performing model/s ranked by the weight of the Akaike Information Criteria corrected for small sample sizes (wAICc); ${R}_{m}^{\mathbf{2}}$: marginal *R*^2^; ${R}_{c}^{\mathbf{2}}$: conditional *R*^2^ with *R*^2^: variance explained; High effect: predictors with the highest effect size; Pred *N*: range of predicted fish abundance and the respective standard error (Pred se *N*); CVerror: cross-validation error and its percentage (CVerror(%)); Val_*R*^2^: results of the direct validation of the observed abundances versus the predicted values for the same locations and the respective *p*-value. All scenarios resulted in some *wAICc* support for the null model. Italics indicate non-significant correlations between the observed values at NR and transferred predictions from the GBR based on a *p*-value <0.05. A total of 133 sites were considered in the GBR.

Scenario	A	B	C	D	E	F	G
Observed *N*	59–2,127	108–2,826	54–1,413	110–654	55–827	17–472	29–2,945
Top models	7	7	7	7	7	7	7
	5			3	3	4	4
*wAICc*	0.342	0.803	0.805	0.493	0.488	0.362	0.355
	0.252			0.260	0.261	0.325	0.331
${R}_{m}^{\mathbf{2}}$	17.3	19.0	18.9	21.5	21.3	21.0	21.2
	12.4			13.4	13.3	23.2	23.4
${R}_{c}^{\mathbf{2}}$	99.0	99.4	98.8	99.3	98.7	98.7	99.4
	99.0			99.4	98.8	98.8	99.4
Highest effect	Depth/NO_3_	NO_3_/Si	NO_3_	NO_3_	NO_3_	Crbnt	Crbnt
			Si				
**Prediction to the GBR**							
Pred *N*_*t*_	561–1,415	444–2,904	222–1,447	493–3,113	248–538	173–958	345–1,914
Pred se *N*_*t*_	0.05–0.2	0.06–0.4	0.06–0.4	0.07–0.34	0.07–0.34	0.07–0.27	0.07–0.27
CV_error_	100.4 ± 31.9	99.2 ± 28.7	53.1 ± 14.2	157.4 ± 55.1	80.6 ± 26.2	51.7 ± 11.8	119.5 ± 21.0
CV_error(%)_	12.8 ± 3.4	13.0 ± 5.3	14.0 ± 6.0	15.8 ± 2.6	19.5 ± 4.2	16.1 ± 6.2	22.5 ± 8.9
Val_*R*^2^	13.0	9.0	9.0	13.6	13.6	18.5	18.6
*p*-value	<0.001	0.002	0.002	<0.001	<0.001	<0.001	<0.001
**Transferred prediction to NR**							
Pred *N*_*t*_	*0–729*	*0–589*	*0–294*	*0–650*	*0–327*	*0–365*	*0–734*
Pred se *N*_*t*_	*0–0.2*	*0–0.27*	*0–0.3*	*0–0.23*	*0–0.2*	*0–0.2*	*0–0.2*
Val_*R*^2^	*<0.1*	*0.2*	*0.2*	*<0.1*	*<0.1*	*<0.1*	*<0.1*
*p*-value	*0.937*	*0.799*	*0.796*	*0.892*	*0.894*	*0.586*	*0.578*

**Table 3:** Results from the Great Barrier Reef (GBR) scenarios predicting fish abundance by fish family to the GBR. Results are summarised for all scenarios for each family unless otherwise specified by splitting results by rows and using letters A–G to identify scenarios. Observed *N*: observed fish abundance; Best model: the best performing model/s ranked by the weight of the Akaike Information Criteria corrected for small sample sizes (wAICc); ${R}_{m}^{2}$: marginal *R*^2^; ${R}_{c}^{2}$: conditional *R*^2^ with *R*^2^: variance explained; Highest effect: predictors with the highest effect size; Pred *N*: range of predicted fish abundance; CVerror: cross-validation error; Val_*R*^2^: results of the direct validation of the observed abundances versus the predicted values for the same locations and the respective *p*-value indicated with asterisks: <0.001 (***), <0.01 (**) and <0.05 (*). Scenario F for Lethrinidae resulted in highest *wAICc* support for the null model and it is not shown. Italicised text: results where direct validation with values observed in the GBR was non-significant. For details on predictors with highest effect refer to Table 1.

Family	Observed *N*	Best model	*wAICc*	${R}_{m}^{2}$	*Rc*^2^	Highest effect	Pred N	CV_error_	Val_*R*^2^
Acanthuridae	0–432	2	0.739–0.861	73.7–80.0	96.4–98.7	Barrier	1–135	4.9 ± 1.9– 25.9 ± 7.5	–50.6^***^
Chaetodontidae	0–163	ADE: 7	0.521–0.684	31.5–36.5	77.6–90.4	SST^2^/PO_4_	12–84	4.5 ± 0.9– 9.8 ± 2.6	25.1–29.5^***^
		BC: 10	0.772–0.855	20.4–22.3	76.5–87.8	SST^2^	14–51	3.2 ± 0.6– 5.8 ± 1.6	22.1^***^
		FG: 8	0.608–0.689	20.1–24.5	71.2–86.5	S	9–35	3.9 ± 1.5– 7.3 ± 1.8	34.7–36.1^***^
Labridae	0–44	ABCDE: 4	0.799–1.000	15.5–39.0	41.1–69.3	PO_4_/Gravel	3–21	1.8 ± 0.3– 4.2 ± 1.2	11.5–23.5^**^
		FG: 8	0.912–0.957	19.5–30.7	42.0–65.2	S	5–22	2.1 ± 0.5– 3.9 ± 0.9	9.4–9.7^*^
Lethrinidae	0–27	ABC: 10	0.614–0.885	9.1–22.5	21.3–52.1	PO_4_/SST	2–6	0.8 ± 0.2– 1.3 ± 0.4	9.9–18.7^***^/^**^
		*DEG: 10*	*0.305–0.681*	*9.5–13.0*	*34.3–61.6*	*PO_4_/SST/* *Barrier*	*2–4*	*0.9 ± 0.3–* *2.2 ± 0.9*	*<6*
Lutjanidae	0–101	ABCDFG: 2	0.465–0.869	40.1–47.8	71.4–86.9	Coast	2–29	2.6 ± 0.9– 5.2 ± 2.4	31.1–65.6^***^
		E: 7	0.556	31.0	64.7	Coast	2–23	2.9 ± 1.0	70.7^***^
Pomacentridae	18–3,561	ABCDE: 3	0.423–0.712	7.6–15.3	98.8–99.5	Depth/NO_3_	171–912	42.3 ± 9.6– 148.5 ± 59.8	8.3–17^***^/^**^
		FG: 5	0.699–0.702	21.0–21.3	98.8–99.4	Sand	137–725	45.5 ± 8.7– 112.7 ± 42.3	28.7^***^
Scaridae	0–472	ABCDE: 2	0.709–0.816	50.9–54.3	90.5–96.7	CRBNT/ Barrier	9–118	9.1 ± 5.1– 24.3 ± 10.1	9.5–14.5^***^/^*^
		*FG: 4*	*0.734–0.785*	*33.0–36.7*	*89.1–95.1*	*CRBNT*	*17–93*	*12.1 ± 3.2–* *22.1 ± 3.8*	*<0.2*
Serranidae	0–46	ABCDEF: 10	0.326–0.957	9.1–37.5	30.0–69.9	SST/PO_4_	2–12	0.9 ± 0.2– 2.3 ± 0.7	13.2–36.2^***^/^*^
		*G: 10*	*0.365*	*11.3*	*61.9*	*SST^2^*	*3–5*	*1.7 ± 0.5*	*<4*
Siganidae	0–129	A: 2	0.775	35.9	83.4	Barrier	6–28	3.2 ± 1.2	14.1^***^
		*BCDEG: 10*	*0.464–0.984*	*22.1–50.0*	*69.6–86.7*	*SST^2^/Barrier^2^*	*1–21*	*1.9 ± 0.4–* *5.0 ± 3.1*	*<3*
		F: 10	1.000	47.5	75.1	SST^2^	1–10	2.9 ± 1.4	16.1^***^
Zanclidae	0–13	2	0.494–0.902	27.6–41.3	29.7–61.2	PO4/ Light/Si	1–8	0.7 ± 0.1– 1.3 ± 0.4	33.7–61.5^***^

**Figure 2:**
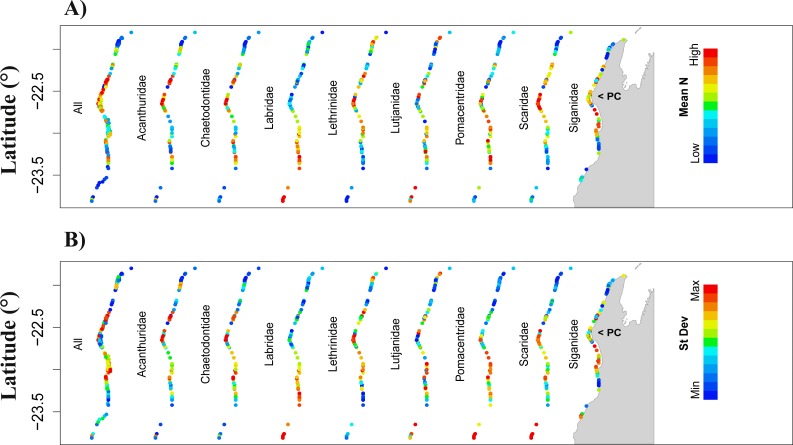
Prediction of total fish abundance (*N*_total_) and fish abundance by fish family (*N*_fam_) at Ningaloo Reef (NR) by the reference NR model. Predictions for locations where predictor values were within the range of values used during model calibration with (A) showing only the mean prediction values for abundance and (B) showing the standard error associated with each mean predicted value. The same colour scheme applies to all maps in each row (for high, low, maximum and minimum prediction values refer to Table 4). Results not shown for fish families for which the null model got highest *wAICc* support (refer to Table 4). Grey area represents the Northwest of Western Australia, longitude: 113.7° to 114.5°E.

Modelling results were generally improved when the data were analysed by fish family. However, for most families, the *wAICc* rank obtained for all GBR scenarios varied and no evident pattern for best scenarios was obtained across all families (Table 3). Acanthuridae and Zanclidae were the only fish families for which all GBR scenarios led to the same *wAICc* results with model 2 (distance to domain boundaries) being the highest ranked for both families (Table 3). Across all fish families, ${R}_{m}^{2}$ was highest for all Acanthuridae scenarios (73.7–80.0%) while cross-validation errors were lowest for Zanclidae (<1.3 ± 0.4%) (Table 3). ${R}_{c}^{2}$ was generally high for all fish families and across all scenarios highlighting high variability within each reef section of the GBR, as was already evident in the Ntotal models. Predictions from most GBR scenarios for all families were positively and significantly correlated with values observed on the GBR and resulted in higher *R*^2^ (e.g., 44.1 ≤*R*^2^ ≤ 50.6%; *p* < 0.001 for Acanthuridae, and 31.1 ≤ *R*^2^ ≤ 70.7%; *p* < 0.001 for Lutjanidae). Exceptions included some scenarios for Lethrinidae (D, E and G), Scaridae (F and G), Serranidae (G) and Siganidae (B, C, D, E and G). Cross-validation errors were generally low (<10%) but ∼25% for Acanthuridae and Scaridae, and >100% for Pomacentridae (Table 3). Predictions for each GBR scenario are presented in Fig. 1.

### Models for NR (*N*_total_ and *N*_fam_)

When modelling *N*_total_ for NR, model 7 (nutrients) also got the highest *wAICc* support, explaining 16.4% of the deviance (Table 4), and the predictor with greatest effect was also *NO*_3_. Predicted *N*_total_ was highest around Point Cloates (central area of NR) (Figs. 2A and 2B—*All*) however, the cross-validation error was high (49.3 ± 21.2%) and model-averaged predictions were unrelated to observed values in NR (*R*^2^ = 9.3% and *p* = 0.108) (Table 4). The test with the additional model including a quadratic term for the predictor *rugosity* ranked the highest (*wAICc* = 0.882) and explained 20.6% of deviance, followed by model 7 (*wAICc* = 0.108, DE% = 16.4) (Table S1). Rugosity also had a large effect jointly with NO_3_, however, the cross validation error was still ∼50%.

For the *N*_fam_ models for NR, highest *wAICc* support also varied among models and fish families. It was highest for model 7 (nutrients) for Acanthuridae and Chaetodontidae, model 3 (physical predictors) for Lutjanidae, and model 10 (temperature) for Siganidae. However, for other families, *wAICc* support was below 0.5 for all models. Generally, most of the deviance was explained by the full model (model 1) (e.g., >23% for Acanthuridae and Chaetodontidae) except for Lutjanidae where model 3 explained most of the deviance (26.5%; Table 4). The most common predictors with biggest effect sizes were *NO*_3_ and *O*_2_, notably for Acanthuridae, Chaetodontidae, Pomacentridae and Scaridae (Table 4). Predicted ranges of fish abundances to NR were always narrower than the observed range, and resulted in high cross-validation errors (>>50%) but were lowest for Labridae (∼52.2%) (Table 4, Fig. S4) possibly associated with the limited amount of data available for NR to detect large-scale patterns, i.e., those not associated with fine-scale changes in the habitat. Direct validation of predicted abundances against observed values in NR resulted in positive significant correlations only for Chaetodontidae (*R*^2^ = 37.1%, *p* < 0.001). For this family, higher predicted abundances were concentrated in the central area of NR (Figs. 2A and 2B). The test with the additional model including a quadratic term for rugosity in NR was ranked highest for five fish families: Chaetodontidae, Labridae, Lethrinidae, Pomacentridae and Scaridae. Rugosity generally had a large effect size in these tests (Table S1), however, similarly to the model of *N*_total_ cross-validation errors were of the same order of magnitude as those obtained without rugosity in the model set (Table S1).

**Table 4:** Results for the models predicting total fish abundance (*N*_total_) and abundance by fish family (*N*_fam_) for Ningaloo Reef (NR). Observed *N*: observed fish abundance; Top model: the best performing model/s ranked by the weight of the Akaike Information Criteria corrected for small sample sizes (*wAICc*); *R*^2^: variance explained; High effect: predictors with the highest effect size; Pred *N*: range of predicted fish abundance and the respective standard error (Pred se *N*); CV_error_: cross-validation error and its percentage (CV_error(%)_); Val_*R*^2^: results of the direct validation of the observed abundances *versus* the predicted values for the same locations and the respective *p*-value. Models for the fish families Serranidae and Zanclidae resulted in high *wAICc* support for the null model and therefore results are not shown. Underlined wAICc values indicate highest ranked models in the model set received *wAICc* >0.5. Italicised text: values for which non-significant correlations (i.e., *p*-value >0.05) for the direct validation of observed *versus* predicted abundance were obtained. A total of 81 sites were used in NR.

Family:	*N*_**total**_	Acanthuridae	Chaetodontidae	Labridae	Lethrinidae	Lutjanidae	Pomacentridae	Scaridae	Siganidae	
Observed *N*	129–1,855	0–579	0–146	31–358	0–41	0–35	11–623	0–450	0–215	
Top model	7	7	7	5	1	3	7	8	10	
			9	2	2		1	7	2	
*wAICc*	0.916	0.957	0.690	0.222	0.339	0.993	0.284	0.429	0.607	
			0.200	0.199	0.220		0.222	0.247	0.238	
*R*^**2**^	16.4	20.5	20.2	7.3	19.9	26.5	7.0	10.2	16.3	
			16.0	14.1	14.0		16.0	9.1	18.7	
High effect	NO_3_	NO_3_	Chl*a*/NO_3_	Sand/Mud/Crbnt	Coast	Depth	NO_3_/Barrier	NO_3_/ O_2_	SST^2^	
**Prediction to NR**										
Pred *N*	*282–187*	*7–89*	6–7	*104–25*	*2–8*	*1–*	*265–499*	*40–164*	*3–37*	
Pred se *N*	*42.0–17.3*	*3.9–2.5*	1.8–17.1	*8.8–109.4*	*0.7–5.3*	*0.4–.7*	*35.6–1.2*	*10.5–8.4*	*1.4–27.5*	
CV_error_	*304.5 ± 83.4*	*77.6 ± 31.0*	27.4 ± 6.9	*54.7 ± 15.9*	*3.9 ± 1.6*	*2.4 ± 1.1*	*221.9 ± 67.7*	*74.9 ± 21.2*	*14.3 ± 7.4*	
CV_error(%)_	*49.3 ± 21.2*	*393.3 ± 337.3*	487.8 ± 374.2	*52.2 ± 19.3*	*178.8 ± 64.2*	*132.7 ± 39.4*	*100.02 ± 110.1*	*905.3 ± 930.0*	*613.3 ± 213.1*	
**Direct validation: observed*versus*predicted abundance**										
Val_*R*^**2**^	*9.3*	*3.3*	37.1	*3.6*	*12.9*	*6.6*	*8.7*	*5.3*	*12.9*	
*P*-value	*0.108*	*0.358*	<0.001	*0.331*	*0.061*	*0.187*	*0.129*	*0.236*	*0.060*	

**Table 5:** Comparison of reference and transferred modelling results. Comparison of results between the reference NR model and the transferred models from the Great Barrier Reef (GBR) predicting fish abundance by fish family (*N*_*fam*_) to Ningaloo Reef (NR). The observed fish counts in NR are included in the first row to assist comparison, and the prediction results obtained by the NR model in the second row (italic values indicate non-significant validation of the reference NR predictions). Results not shown for Serranidae and Zanclidae due to the higher *wAICc* support obtained for the null model in the NR reference models. Results for GBR scenarios are only shown where we obtained significant direct validation with values observed in the GBR and low *wAICc* support (≤0.1) for the null model. Bold font indicates best values in each section for each fish family. Underlined values for Chaetodontidae indicate the only fish family for which the NR models resulted in significant correlations between predicted abundance and observed values.

Scenario		Acanthuridae	Chaetodontidae	Labridae	Lethrinidae	Lutjanidae	Pomacentridae	Scaridae	Siganidae
Observed *N*_fam_		0–579	0–146	31–358	0–41	0–41	11–1,623	0–450	0–215
NR Pred *N*_fam_ sd		*5–171*	6–66	*104–225*	*2–8*	*1–4*	*265–99*	*40–164*	*3–37*
		*1.2–4.2*	1.8–17.1	*8.8–109.4*	*0.7–53.3*	*1.1–1.7*	*35.6–91.2*	*10.5–48.4*	*1.4–27.5*
Range of predictions	A	0–100	0–3,913	0–22	0–266	6–15	0–595	0–100	**0–34**
	B	**0–136**	0–1,125	0–22	0–122	4–15	0–629	**0–115**	
	C	0–67	0–423	0–12	0–25	3.0–8.5	0–315	0–57	
	D	0–104	0–1,199	**0–24**		12–58	0–605	0–96	
	E	0–51	0–624	0–13		9–34	0–303	0–48	
	F	0–67	0–16	0–8	**0–5**	3–8	0–383		0 –4
	G	0–132	**0–28**	0–15		3–12	**0–767**		
Abs difference/(percentage)	A	106.2 ± 54.1 (96.5 ± 4.4%)	237.9 ± 505.6 (763.7 ± 1,819.3%)	120.3 ± 29.2 (88.6 ± 6.7%)	18.4 ± 35.7 (590.5 ± 1,307.9%)	8.1 ± 1.8 (464.8 ± 162.5%)	187.7 ±132.1 (46.7±32.4%)	**80.4±38.8** **(69.3 ± 22.3%)**	11.9 ± 8.0 (147.3 ± 140.0%)
	B	105.6 ± 54.1 (95.9 ± 4.7%)	109.2 ± 182.0 (356.9 ± 653.3%)	120.2 ± 29.1 (88.6 ± 7.2%)	10.9 ± 18.3 (336.2 ± 672.2%)	5.8 ± 2.3 (328.2 ± 149.9%)	**177.5 ± 128.5** **(43.4 ± 30.0%)**	86.2 ± 36.8 (75.0 ± 19.5%)	
	C	106.8 ± 54.2 (97.4 ± 3.1%)	44.8 ± 62.3 (141.3 ± 232.7%)	127.3 ± 29.1 (93.9 ± 3.8%)	4.1 ± 3.7 (105.4 ± 136.7%)	3.2 ± 1.3 (185.6 ± 89.2%)	210.1 ± 118.0 (49.9 ± 26.5%)	98.4 ± 31.5 (87.0 ± 10.1%)	
	D	106.9 ± 54.1 (97.6 ± 3.1%)	103.6 ± 184.4 (333.9 ± 664.6%)	**119.7 ± 28.4** **(88.2 ± 7.5%)**		15.9 ± 4.2 (919.9 ± 397.4%)	200.4 ± 132.3 (49.8 ± 32.4%)	85.2 ± 37.1 (74.1 ± 19.6%)	
	E	107.2 ± 54.2 (97.8 ± 2.7%)	60.1 ± 93.8 (189.3 ± 344.6%)	126.8 ± 29.0 (93.6 ± 4.1%)		9.9 ± 2.4 (571.2 ± 239.9%)	211.8 ± 127.3 (50.3 ± 29.3%)	97.7 ± 31.6 (86.4 ± 10.2%)	
	F	105.3 ± 54.2 (95.4 ± 5.2%)	25.3 ± 12.8 (68.9 ± 24.0%)	130.2 ± 29.9 (96.0 ± 2.7%)	**2.6 ± 1.8** **(52.6 ± 26.3%)**	**2.4 ± 1.1** **(139.6 ± 75.8%)**	230.0 ± 124.4 (55.2 ± 27.7%)		11.1 ± 7.5 (89.0 ± 18.3%)
	G	**103.4±54.2** **(93.2 ± 7.6%)**	**18.6 ± 14.1** **(55.3 ± 55.4%)**	126.4 ± 30.3 (96.1 ± 4.7%)		3.7 ± 1.8 (208.6 ± 113.6%)	200.4 ± 136.9 (47.1 ± 30.8%)		
% grid–cell ≤ 15%	A	0.0	13.0	0.0	5.2	0.0	**15.6**	0.0	**10.4**
	B	0.0	7.8	0.0	**10.4**	0.0	**15.6**	0.0	
	C	0.0	6.5	0.0	9.1	1.3	2.6	0.0	
	D	0.0	**18.2**	0.0		0.0	11.7	0.0	
	E	0.0	5.2	0.0		0.0	2.6	0.0	
	F	0.0	2.6	0.0	9.1	**3.9**	7.8		1.3
	G	0.0	13.0	0.0	–	2.6	11.74		
% high-versus-low	A	0.0	**96.1**	0.0	**98.7**	0.0	11.7	1.3	**42.9**
	B	5.2	0.0	0.0	1.3	1.3	0.0	**89.6**	
	C	1.3	0.0	0.0	1.3	2.6	0.0	0.0	
	D	**88.3**	0.0	14.3		**53.2**	28.6	5.2	
	E	0.0	0.0	**57.1**		28.6	**41.6**	0.0	
	F	1.3	0.0	0.0	1.3	0.0	0.0		16.9
	G	3.9	3.9	28.6		14.3	18.2–		

**Figure 3:**
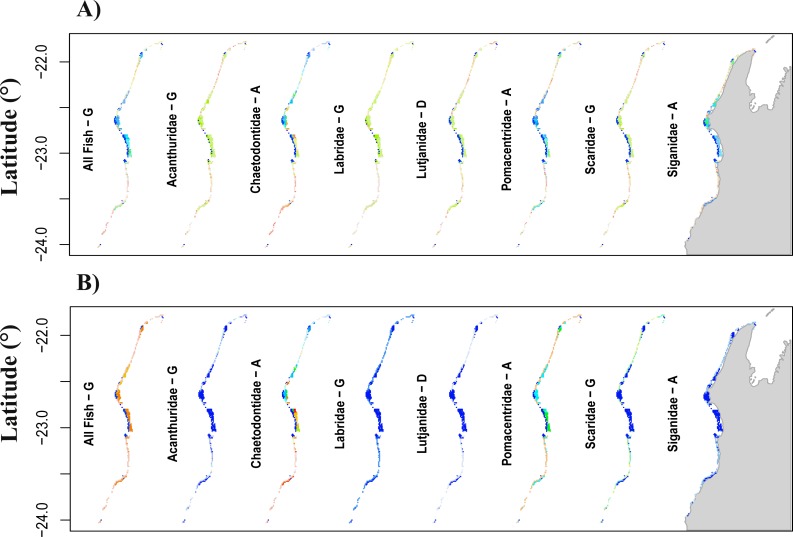
Prediction of total fish abundance (*N*_total_) and fish abundance by fish family to Ningaloo Reef (NR) by the transferred models from the GreatBarrier Reef (GBR). Results for each family shown for the scenario resulting in best ‘*%high-versus-low*’ indicated with letter from (A to G) as per Table 5 (refer to Fig. S4 for predictions from all the transferred GBR scenarios to NR). Colour scheme shown in Fig. 1 applies here with: (A) showing maximum and minimum values for each map independently, and (B) reflecting ranges of values across all maps. For predicted minimum and maximum abundance values for each scenario refer to Tables 2 and 4. Grey area represents the Northwest of Western Australia, longitude: 113.7° to 114.5°E.

### Models transferred from the GBR to NR

When transferring the *N*_total_ models from the GBR to NR, the range of predicted abundances was always narrower than that observed at NR (Tables 2, 4 and 5) and we obtained no significant correlations between observed and predicted values (Fig. 3). Similar results were obtained for predictions from the transferred *N*_fam_ models from the GBR to NR, which resulted in low, non-significant *R*^2^ (<6%, *p* >0.05) when directly compared with observed values at NR. Also, none of the transferred models (downscaled or not) resulted in prediction ranges close to the observations at NR (Tables 3 and 5). Absolute differences between transferred and reference NR predictions were generally high for all scenarios and fish families, but lowest for Chaetodontidae, Lethrinidae and Pomacentridae (<55.3%) (Table 5).

Transferred predictions for Chaetodontidae, the only family for which the NR model resulted in a significant correlation between predictions and observed values, resulted in the highest percentage of grid cells with values differing by ≤15% from those obtained by the reference model (Chaetodontidae: 18.2% for scenario D, and 13% for both scenarios A and G) (Table 5). Comparable rescaled patterns between transferred and reference NR predictions were obtained for this fish family (96.1%; scenario A) (Table 5 and Fig. 3 for comparison Fig. 2).

## Discussion

Readily available models that reliably predict fish abundances would have the potential to assist management and conservation of marine ecosystems (Knowlton et al., 2010; Bejarano, Mumby & Sotheran, 2011; Plaisance et al., 2011). Following recent success in transferring predictive models of fish species richness between coral reefs (Sequeira et al., 2016), we assessed here the transferability of predictive models of reef fish abundances between the same two coral reef systems and using the same set of environmental predictors. Due to the relevance of fish abundance models for stock assessments (Bejarano, Mumby & Sotheran, 2011), we specifically developed models of total abundance and of abundance by fish family to compare transferability results when the response variable does and does not include exploited species. Model transferability from the GBR to NR varied among the response variables tested (i.e., for *N*_total_ and *N*_fam_) with no obviously superior model emerging for the GBR scenarios tested.

Predicting fish abundance for each system individually was challenging but generally improved when considering fish families separately. For example, our *N*_fam_ models for most GBR scenarios resulted in greater and significant *R*^2^ for all families, including the exploited Lutjanidae for which predictions resulted in the highest *R*^2^ (70%). This result highlights that good predictive models of abundance by fish family including exploited species is achievable, and that predictions will be useful to understand patterns in abundance. This improvement when considering fish families separately was also verified for NR, where models of Chaetodontidae abundance were of moderate goodness-of-fit. Chaetodontidae are of little commercial interest, and are usually sedentary (site-attached) occurring as single individuals or in pairs. These factors may reduce survey bias in estimating their abundances, contributing to the better performance of the NR models despite the low number of sampling locations considered. However, with limited availability of fish abundance data at the scale of the entire NR, most models resulted in weak, non-significant correlations with observed abundances in that system (Table 2).

Due to the general lack of significant modelling results at NR, we could only directly assess and compare the transferred and reference models’ results for Chaetodontidae. For this fish family, we found small absolute differences between the transferred and reference predictions and also a high similarity in the predicted patterns of low and high abundance (i.e., *%low-versus-high* predictions). With Chaetodontidae including highly sedentary and territorial species, our results suggest that local-scale processes of relevance to these species might be well captured by the variables in our models, whereas the possible reasons behind the lack of transferability for other fish families deserves further investigation. While the focus here was to understand what is required to transfer models successfully, application of these findings are also likely to be valuable for families including commercial species. Overall, our results indicate that similarities between predictions from transferred and reference models of fish abundance may be achievable. Transferring a model of abundance could, therefore, be useful where data are insufficient to build a new reference model for that location. However, at this point, we were unable to identify a consistent approach to transferring these models that would give confidence in other similar applications.

Our inability here to construct consistently transferable models of abundance should not be taken as failure, but rather as an opportunity to begin learning about how transferable models can be built, and ultimately, their utility improved. For example, the importance of nitrate (NO_3_) as a predictor suggests that productivity might be an important habitat characteristic that contributes to determine abundance in these reef systems (Barneche et al., 2016). Therefore, if available, related predictors could be included to try and improve the models and their transferability. Indeed, the results of our models highlight that a major challenge in understanding and improving model transferability lies first in obtaining consistently robust predictions of fish abundances for the reference and target locations. Therefore, a useful way forward might be to first construct better, and more ecologically meaningful, predictive models of abundance than attempting the construction of more transferable ones.

To assist model improvement, where available, inclusion of variables that better represent the biology of reef systems or their benthic communities and habitats (Pittman, Costa & Battista, 2009; Yates et al., 2016) should be considered. As shown here for the NR models with the inclusion of *rugosity* (which in coral reefs is a largely biogenic variable), local, fine-scale topographic predictors might considerably improve model fit. If such predictors, more directly associated with patterns of fish abundances, were available for the GBR as well, it is plausible that better model transferability would have been obtained. Our contention here is supported by previous studies showing that models using different abundance-related response variables have different data needs, such as levels of replication (sensu Jones et al., 2015). In turn, these needs will depend, for example, on the variability of fish abundances, as these can be strongly influenced by many factors, such as recruitment, predation or fishing, i.e., demographic stochasticity (Sale & Douglas, 1984), or ecosystem biodiversity and human impacts (Ridgway, Dunn & Wilkin, 2002), with better models expected for fish species with more stable abundances (Jones et al., 2015).

The fish abundance data used here resulted from the application of different survey designs targeting different habitats in each reef system. It included the exposed side of the reef slope with greater coral cover on the GBR (Sweatman et al., 2008), while at NR, most samples were taken on reef flat areas with less three dimensional structure. Because greater structural complexity (i.e., high coral cover) can promote greater abundances of reef fishes (e.g., Gratwicke & Speight, 2005), this difference in the habitats sampled on the GBR and NR may have hindered the transferability of the GBR models to NR. Results for Zanclidae are a good example. Being a monospecific family with low population densities, predictions for this species were consistent across all GBR scenarios considered, while with the NR reference model *wAICc* support was greatest for the null model. This result is in agreement with this species’ occurrence on reef flats, which includes much lower densities than in other habitats. Therefore, targeting equivalent habitats at the two locations may also assist model transferability, but direct tests will be required to better understand the magnitude of such an effect.

Another possible reason for the lack of model transferability observed here might be the spatial heterogeneity of the habitats in both reef ecosystems, as both geographical and geomorphological drivers affect the distribution of reef fish species (Pinca et al., 2012). Moreover, some species groups vary among habitats within the GBR. For example, the cross-shelf patterns of herbivores can be markedly different between inner-, mid- and outer-shelf reefs (Wismer, Hoey & Bellwood, 2009). When testing model transferability in space, observations for the same set of predictors also need to be available for both study systems. Here we used environmental and spatial predictors available around Australia to maximize the number of predictors that could be included and tested in our model set. Even still, this information was only available at a coarse resolution, and the results we report here may contrast with those that would have been obtained from finer resolution predictors such as roughness (e.g., estimated by acoustic methods) and fishing pressure that can affect fish abundances by removing individuals and destabilizing population dynamics (Hsieh et al., 2006; Anderson et al., 2008; Bejarano, Mumby & Sotheran, 2011; Rana et al., 2014).

To test the transferability of predictive models of fish abundances between two widely separated coral reefs, we constructed models that used a similar same set of predictors and scenarios previously shown to produce transferable models for fish species richness (Sequeira et al., 2016). The poor transferability of our abundance models compared to those developed for species richness highlights potential dissimilarities between the drivers of richness and abundance or in the way different ecological processes are captured by the predictors used and translated into this response variable. In particular, spatial predictors contributed to the transferability of the species richness models applied to the same two distant reefs (Sequeira et al., 2016) but were much less important when predicting fish abundances, with the exception of models for Acanthuridae and Zanclidae on the GBR. Interestingly, Acanthuridae were also previously shown to be an exception in beta-diversity stability relationships across fish families, potentially because of the way they respond to temporal variation in macro-algal availability (Mellin et al., 2014). Such differences in model transferability suggest that species richness patterns are more stable and better explained at large spatial scales (Caley & Schluter, 1997) than fish abundances, and models may therefore be more transferable for species richness. Conversely, fish abundances in marine systems are notable for their variability (Sale & Douglas, 1984; Caley et al., 1996) that might diminish model transferability. Generally, the predictors we used explained more variance in fish species richness than in fish abundance (Mellin et al., 2010; Sutcliffe et al., 2014) but because changes in abundance may be a more immediate indicator of changes in climate or responses to management intervention, it is also important to find ways to overcome challenges associated with building transferable predictive models of abundances.

Potentially, improvements to both fish abundance models and their transferability could be achieved by using different combinations of predictors (e.g., mixing physical and biological variables in the same model), by using biomass estimates, or by grouping species in different ways, such as, trophic role or ecological function. Here we grouped species by family and then tested for predictability and transferability. However, because species within families might differ considerably in behaviour, territoriality, food requirements, and thus, their resilience to change, other classification schemes such as, by functional group or size spectra, might result in better transferability. Furthermore, due to high abundance variability, relating yearly abundances to lagged effects of disturbance and/or fishing instead of averaging across years might also assist in improving transferability.

## Conclusions

Having tools available that can rapidly and efficiently increase our understanding of marine ecosystems and their capacity to respond to accelerating impacts of anthropogenic disturbances is crucial. Such capacities are particularly important for the most understudied regions, which also host the greatest amount of biodiversity (Fisher et al., 2011b) and understudied taxa (Fisher et al., 2011a). However, the challenges associated with developing such tools can only be overcome through establishing a set of predictors that are more relevant at a local scale across ecosystems leading to a better understanding of the causes of abundance patterns. Ultimately, as such information increases so should our knowledge of how to build transferable predictive models of abundances of fishes and other taxa.

##  Supplemental Information

10.7717/peerj.4566/supp-1 Supplemental Information 1 Summary of test results when including a model with a quadratic term for rugosity in the model set, i.e., Model 13 poly (rugosity,2), for NR predicting total fish abundance (*N*_total_) and abundance by fish family to Ningaloo Reef Shown are the two best performing models according to the weights of the Akaike Information Criteria corrected for small sample sizes (*wAICc*) (Top models) and the respective *wAICc* values and percentage of deviance explained (*DE%*), the predictors with the highest effect sizes (High effect), and the cross-validation error in abundance and its percentage (*CV*_error_ and *CV*_error(%)_, respectively). Models for the fish families Serranidae and Zanclidae resulted in high * wAICc* support for the null model and therefore results are not shown.

10.7717/peerj.4566/supp-2 Figure S1 Location of the sampling sites where fish data were collected in (A) Ningaloo Reef (adapted from Sequeira et al., 2016) and (B) the Great Barrier Reef

10.7717/peerj.4566/supp-3 Figure S2 Ranges of predictor variables: (A) Sampled locations in Ningaloo Reef, and (B) All locations in Ningaloo Reef

10.7717/peerj.4566/supp-4 Figure S3 Latitude degrees are shown in *y*-axis. The longitude in the GBR spans 130 to 140 degrees. For maximum and minimum prediction values refer to Table 2

10.7717/peerj.4566/supp-5 Figure S4 Prediction of total fish abundance (*N*_total_) and fish abundance by fish family to Ningaloo Reef by all the transferred models from the GreatBarrier Reef (i.e., by all scenarios)

10.7717/peerj.4566/supp-6 Data S1 Data tables for the GBR and NR including all the predictors considered as well as the total abundances and abundances by fish family in each reef

## Additional Information and Declarations

### Data Availability

The following information was supplied regarding data availability: The fish count datasets from the Great Barrier Reef and Ningaloo Reef have been provided as Supplemental Files.
