# Supplementary material for: Challenges of transferring models of fish abundance between coral reefs
Source: PeerJ. 2018 Apr 17;6:e4566. doi: 10.7717/peerj.4566 (PMC5909686; doi:10.7717/peerj.4566)
Supplement: Supplemental Information 1 — Shown are the two best performing models according to the weights of the Akaike Information Criteria corrected for small sample sizes (wAICc) (Top models) and the respective wAICc values and percentage of deviance explained (DE%), the predictors with the highest effect sizes (High effect), and the cross-validation error in abundance and its percentage (CVerror and CVerror(%), respectively). Models for the fish families Serranidae and Zanclidae resulted in high wAICc support for the null model and therefore results are not shown. [file peerj-06-4566-s001.docx]

## Table S1: Summary of test results when including a model with a quadratic term for rugosity in the model set, i.e., Model 13 ~ poly(rugosity, 2), for NR predicting total fish abundance (N_total_) and abundance by fish family to Ningaloo Reef.

Shown are the two best performing models according to the weights of the Akaike Information Criteria corrected for small sample sizes (*wAICc*) (Top models) and the respective *wAICc* values and percentage of deviance explained (*DE%*), the predictors with the highest effect sizes (High effect), and the cross-validation error in abundance and its percentage (CV_error_ and CV_error(%)_, respectively). Models for the fish families Serranidae and Zanclidae resulted in high *wAICc* support for the null model and therefore results are not shown.

| **Family:** | ***N_total_*** | **Acanthuridae** | **Chaetodontidae** | **Labridae** | **Lethrinidae** | **Lutjanidae** | **Pomacentridae** | **Scaridae** | **Siganidae** |
| --- | --- | --- | --- | --- | --- | --- | --- | --- | --- |
| **Top models** | 13 | 7 | 13 | 13 | 13 | 3  11 | 13 | 13 | 10 |
|  | 7 | 1 | 7 | 5 | 1 |  | 7 | 8 | 2 |
| ***wAICc*** | 0.882 | 0.921 | 0.994 | 0.890 | 0.651 | 0.993  0.004 | ~1 | 0.519 | 0.629 |
|  | 0.108 | 0.073 | 0.040 | 0.023 | 0.082 |  | <0.001 | 0.157 | 0.178 |
| ***R^2^*** | 20.6 | 20.5 | 30.1 | 17.7 | 12.3 | 27.1  8.6 | 25.6 | 12.3 | 17.1 |
|  | 16.4 | 24.1 | 20.8 | 7.6 | 18.2 |  | 7.9 | 9.9 | 18.9 |
| **High effect** | Rugosity/ NO_3_ | NO_3_/ O_2_ | Rugosity/ Chl*a*/NO_3_ | Rugosity/ Sand/Mud | Dist2Coast/ Rugosity | Depth/ Light | Rugosity/ NO_3_/Dist2Out | NO_3_/ Rugosity | SST^2^/ Rugosity |
| Cross-validation | | | | | | | | | |
| **CV_error_** | 301.0±83.4 | 78.6±27.2 | 27.6±7.4 | 50.6±16.1 | 3.7±1.4 | 2.9±2.2 | 215.8±60.2 | 72.7±22.7 | 14.5±7.2 |
| **CV_error(%)_** | 48.6±20.8 | 368.2±330.6 | 430.6±301.3 | 52.5±19.1 | 143.9±48.4 | 143.9±132.4 | 72.9±29.1 | 992.8±939.2 | 569.9±219.4 |
