# Supplementary figures and images for: Challenges of transferring models of fish abundance between coral reefs
Source: PeerJ. 2018 Apr 17;6:e4566. doi: 10.7717/peerj.4566 (PMC5909686; doi:10.7717/peerj.4566)

A)

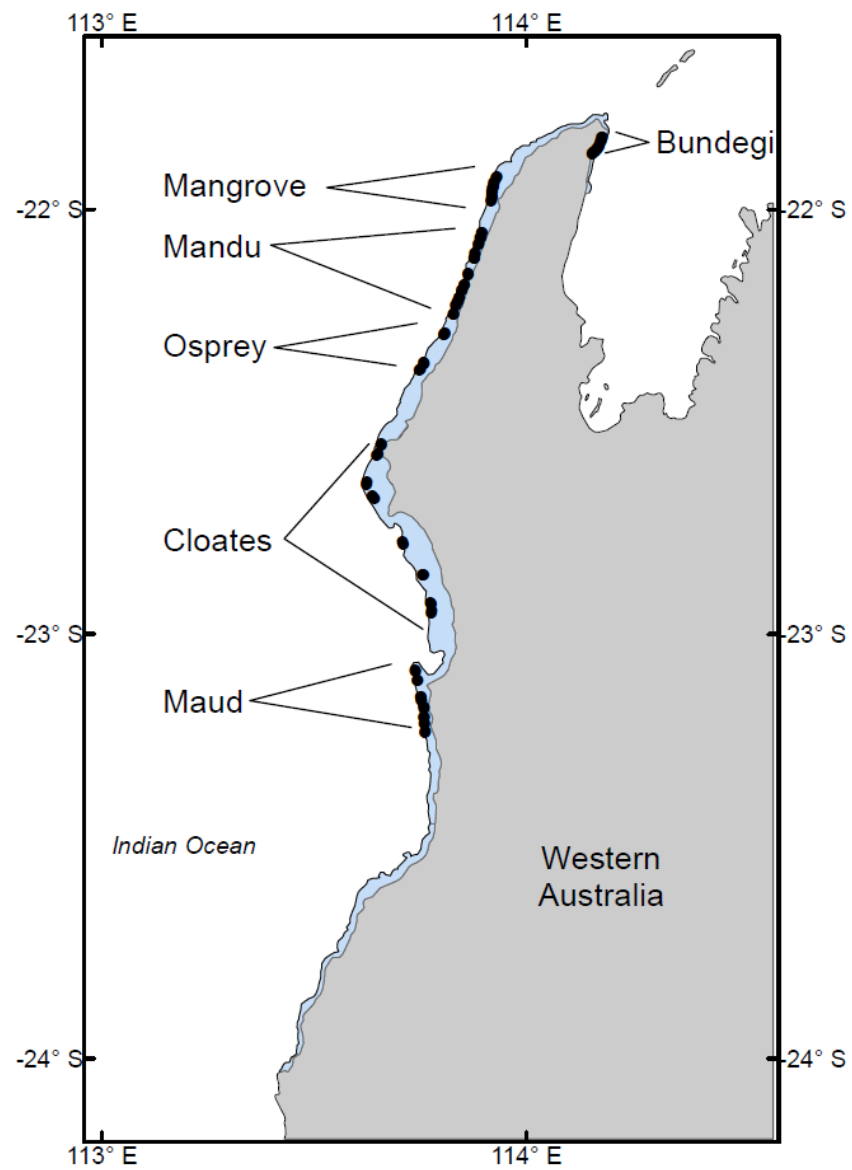

B)

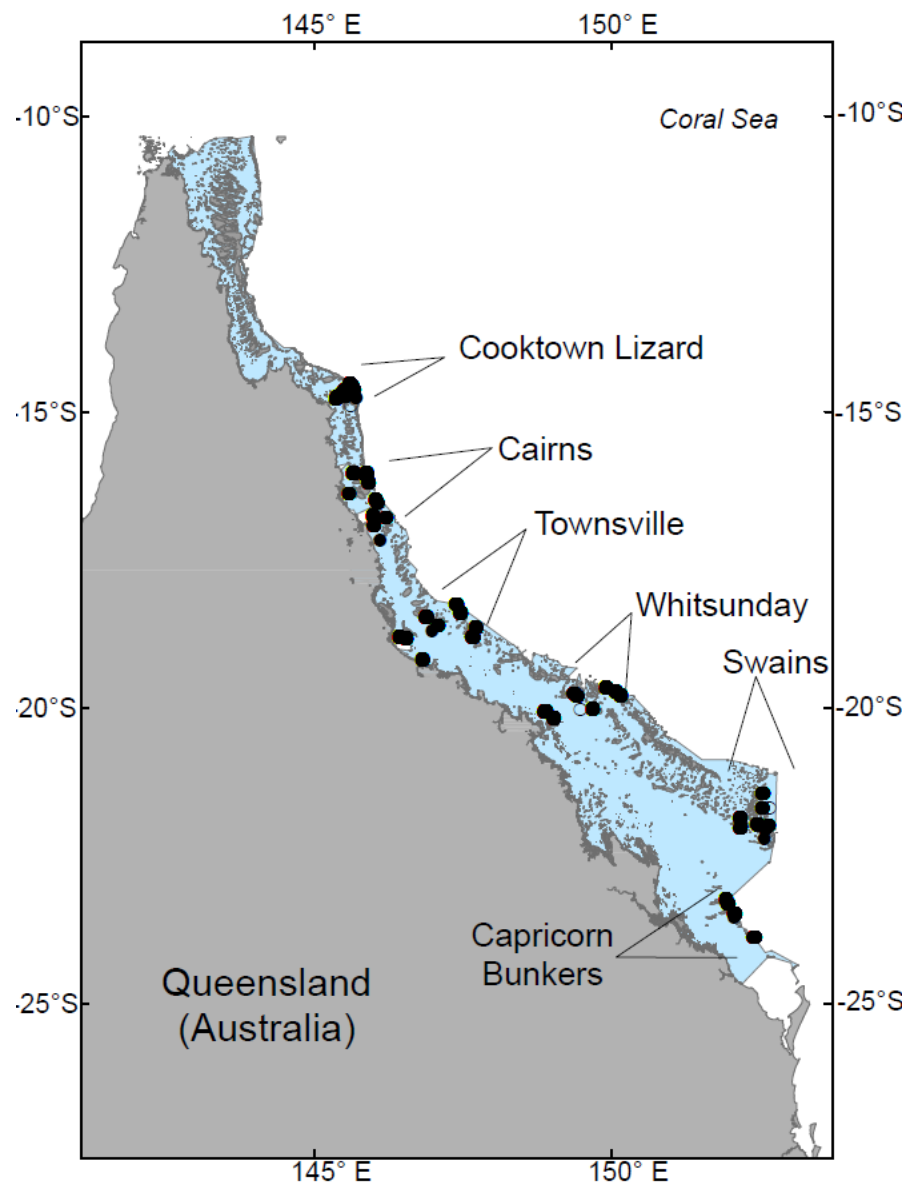

A)

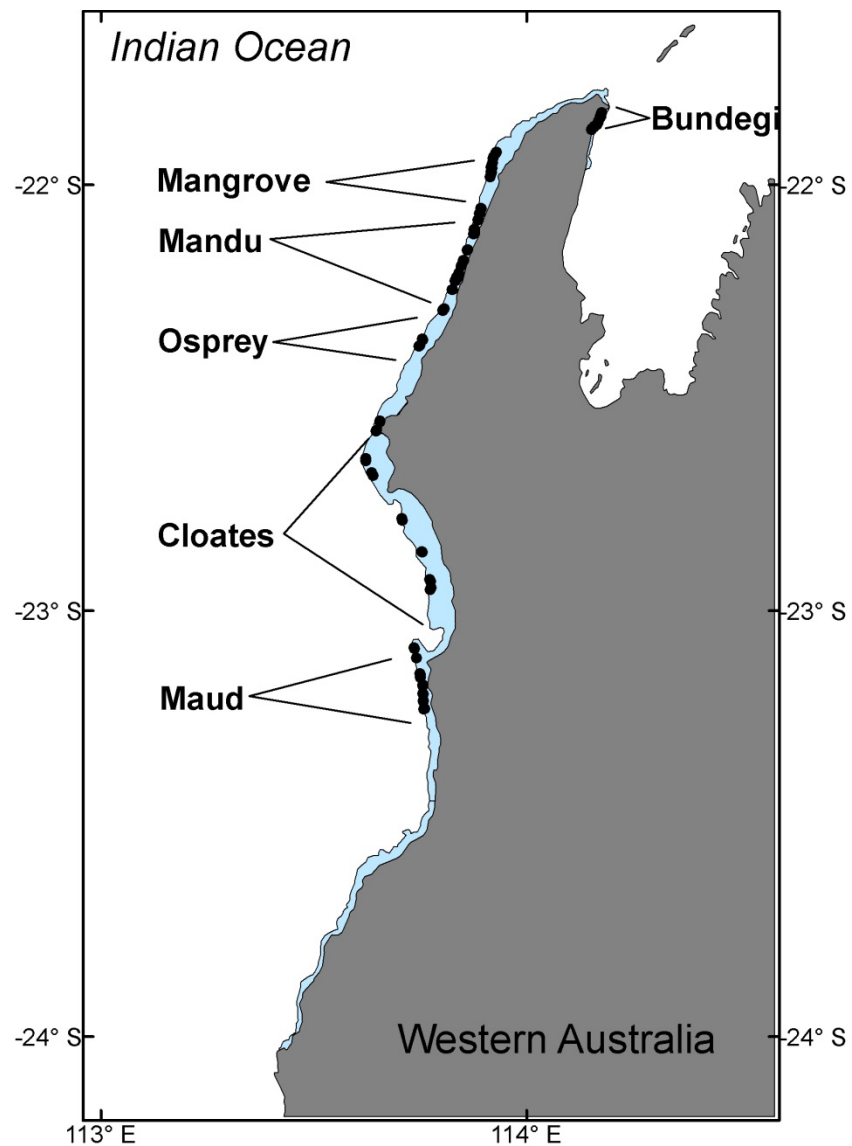

B)

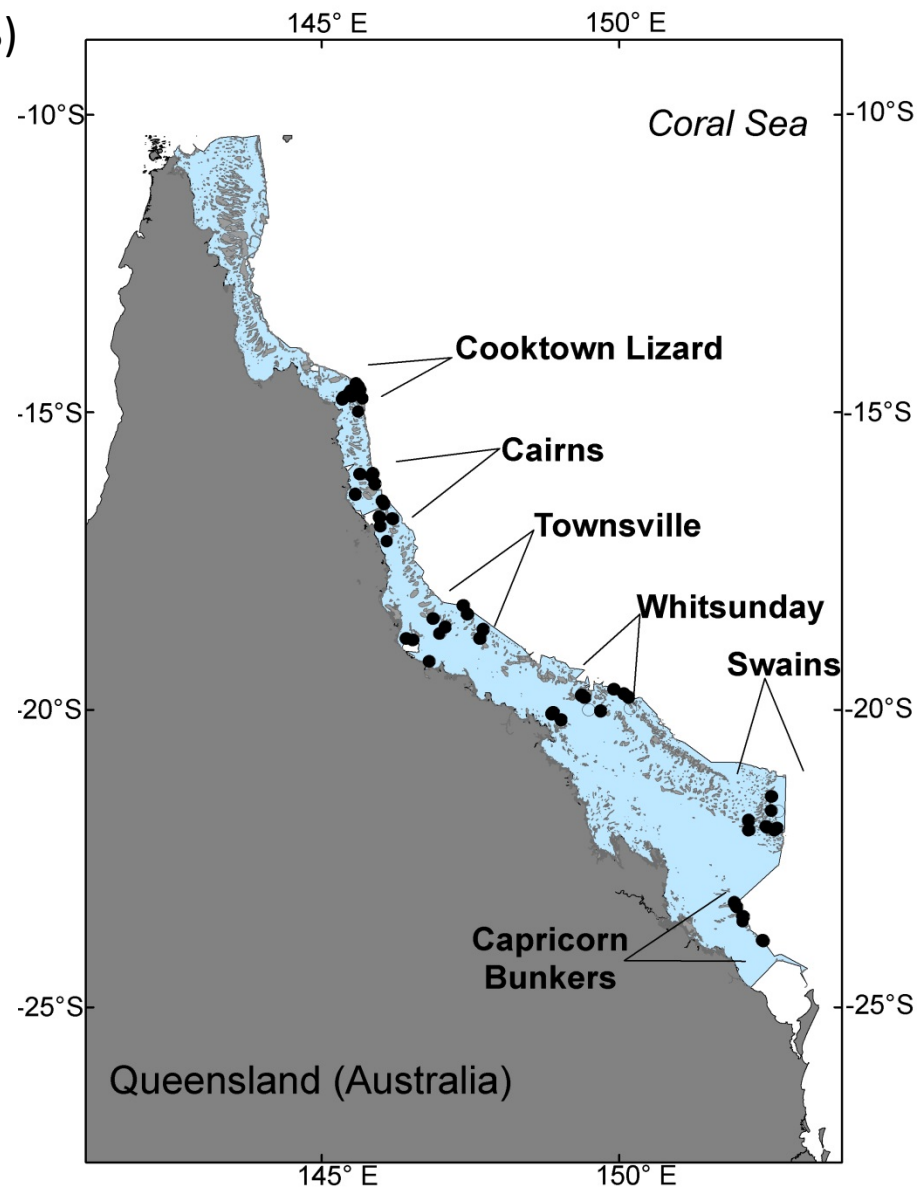

Supplement: Figure S1 [file peerj-06-4566-s002.pdf]

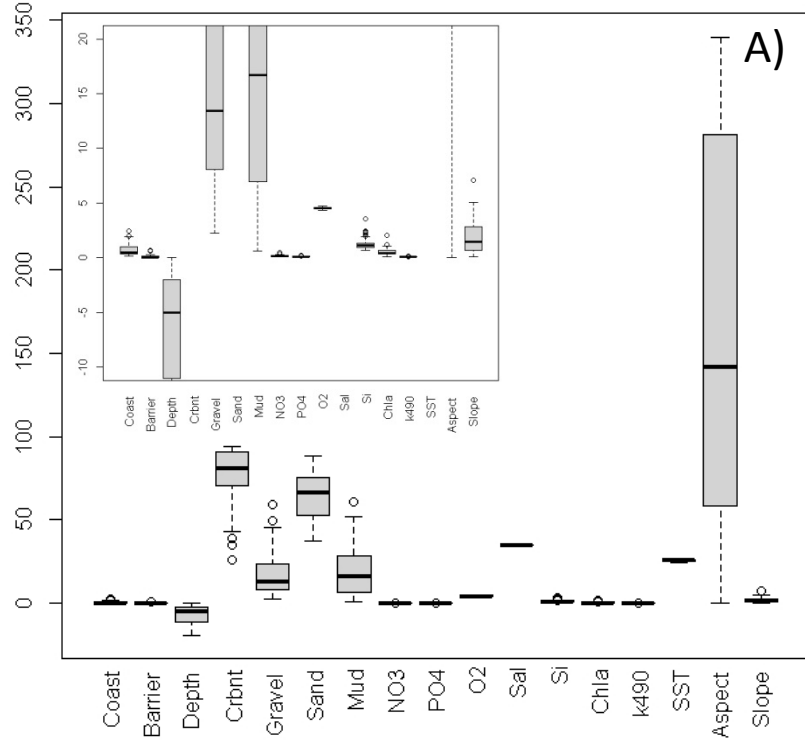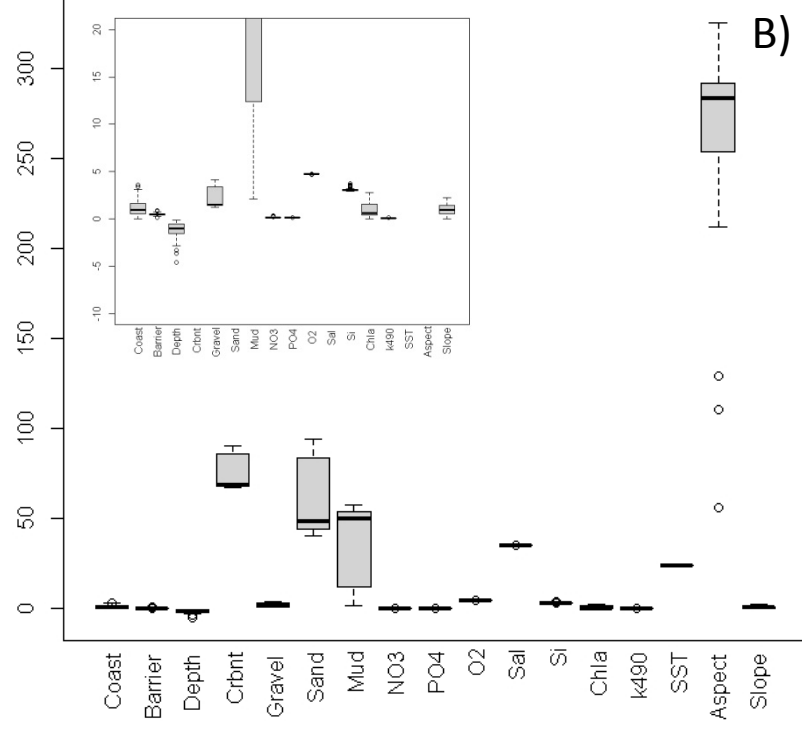

Supplement: Figure S2 [file peerj-06-4566-s003.pdf]
