# Supplementary material for: Challenges of transferring models of fish abundance between coral reefs
Source: PeerJ. 2018 Apr 17;6:e4566. doi: 10.7717/peerj.4566 (PMC5909686; doi:10.7717/peerj.4566)
Supplement: Figure S3 — The longitude in the GBR spans 130 to 140 degrees. For maximum and minimum prediction values refer to Table 2 [file peerj-06-4566-s004.pdf]

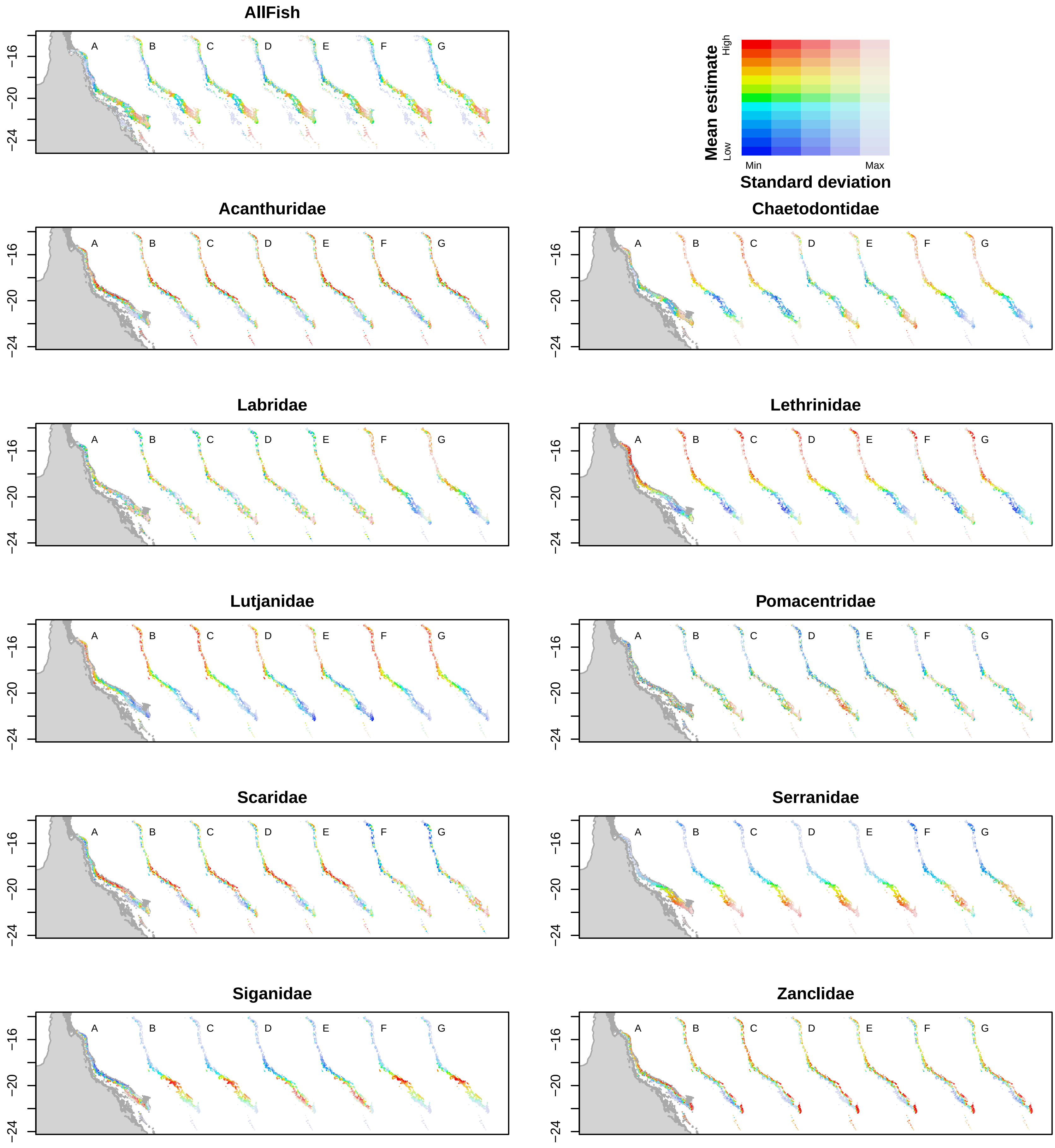
AllFish
Mean estimate
High
Low
Min
Max
Standard deviation
Acanthuridae
Chaetodontidae
Labridae
Lethrinidae
Lutjanidae
Pomacentridae
Scaridae
Serranidae
Siganidae
Zanclidae
A
B
C
D
E
F
G
−16
−20
−24
