# Supplementary material for: Challenges of transferring models of fish abundance between coral reefs
Source: PeerJ. 2018 Apr 17;6:e4566. doi: 10.7717/peerj.4566 (PMC5909686; doi:10.7717/peerj.4566)
Supplement: Figure S4 [file peerj-06-4566-s005.pdf]

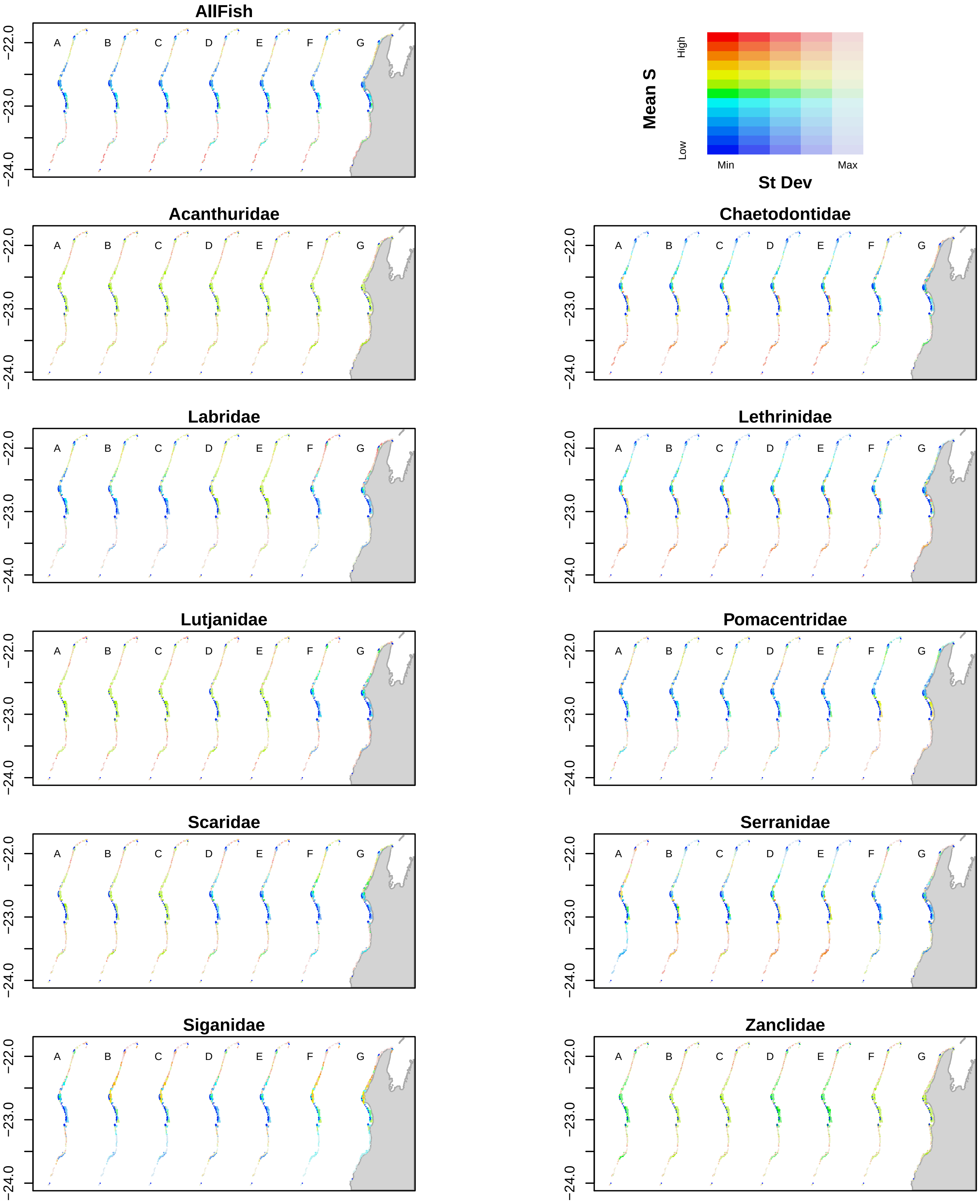
AllFish
Acanthuridae
Chaetodontidae
Labridae
Lethrinidae
Lutjanidae
Pomacentridae
Scaridae
Serranidae
Siganidae
Zanclidae
A
B
C
D
E
F
G
−22.0
−23.0
−24.0
Mean S
High
Low
Min
Max
St Dev
